# Childhood obesity trends from primary care electronic health records in England between 1994 and 2013: population-based cohort study

**DOI:** 10.1136/archdischild-2014-307151

**Published:** 2015-01-29

**Authors:** Cornelia H M van Jaarsveld, Martin C Gulliford

**Affiliations:** Department of Primary Care and Public Health Sciences, King's College London, London, UK

**Keywords:** Obesity, Monitoring, Epidemiology, Growth

## Abstract

**Objective:**

This study aimed to use primary care electronic health records to evaluate the prevalence of overweight and obesity in 2–15-year-old children in England and compare trends over the last two decades.

**Design:**

Cohort study of primary care electronic health records.

**Setting:**

375 general practices in England that contribute to the UK Clinical Practice Research Datalink.

**Patients:**

Individual participants were sampled if they were aged between 2 and 15 years during the period 1994–2013 and had one or more records of body mass index (BMI).

**Main outcome measure:**

Prevalence of overweight (including obesity) was defined as a BMI equal to or greater than the 85th centile of the 1990 UK reference population.

**Results:**

Data were analysed for 370 544 children with 507 483 BMI records. From 1994 to 2003, the odds of overweight and obesity increased by 8.1% per year (95% CI 7.2% to 8.9%) compared with 0.4% (−0.2% to 1.1%) from 2004 to 2013. Trends were similar for boys and girls, but differed by age groups, with prevalence stabilising in 2004 to 2013 in the younger (2–10 year) but not older (11–15 year) age group, where rates continued to increase.

**Conclusions:**

Primary care electronic health records in England may provide a valuable resource for monitoring obesity trends. More than a third of UK children are overweight or obese, but the prevalence of overweight and obesity may have stabilised between 2004 and 2013.

**What is already known on this topic**

- Evidence from international sources suggests that childhood obesity levels may have started to plateau.
- The Health Survey for England and the English National Child Measurement Programme provide evidence on childhood obesity but with small samples or limited age ranges.

**What this study adds**

- Primary care electronic health records in England are suitable for monitoring obesity trends in children.
- More than a third of UK children are overweight or obese, but the prevalence may have stabilised between 2004 and 2013.
- In the older age group (11–15 years), the upward trend was still evident, highlighting the need for interventions to focus on this age group.

## Introduction

Obesity has become an important public health priority.^1^ Several determinants of obesity originate in or before childhood,^2^ ^3^ and obese children may become obese as adults.^4^ Obesity in children has increased dramatically^5^ with important clinical and economic impacts.^6^ Consequently, understanding trends in obesity is important for monitoring population health and informing policy initiatives. Recent reports suggest that the increase in obesity in children may have levelled off since 2000.^7^ ^8^ However, only a few countries have data on younger children (aged under 6 years). Stabilising trends in children are reported for the USA,^9^ ^10^ the Netherlands^11^ and Australia.^12^

In England, childhood obesity prevalence data have been collected in the Health Survey for England (HSE) since 1995,^13^ and in the National Childhood Measurement Programme (NCMP) since 2005.^14^ Both provide important information, but data from the HSE are limited by the small number of children included each year, while the NCMP only includes data for children in reception and year 6 classes. Data from the HSE suggest that the prevalence of overweight and obesity among 2–15 year olds peaked in 2004–2005 and may have declined slightly since.^15^ Data from the NCMP suggest that overweight and obesity prevalence declined between 2006 and 2013 in 4–5 year olds, but increased in 10–11 year olds.^16^

The Clinical Practice Research Datalink (CPRD) is a large database holding the electronic health records of about 7% of UK family practices. The present study aimed to use primary care electronic health records to examine prevalence of overweight and obesity in 2–15-year-old children in England from 1994 to 2013, and to compare trends over two decades.

## Methods

### Study population

Data were obtained from the UK CPRD. The CPRD includes research quality data on 5.5 million currently registered UK primary care participants from over 680 family practices.^17^ The size and geographical distribution of general practices are broadly representative of the UK population, and the validity of data is good.^18^ The protocol was approved by the Independent Scientific Advisory Committee (ISAC 13-194). The sample was restricted to 375 general practices in England that participate in the data linkage scheme. Individual participants were sampled if they were aged between 2 and 15 years during the period 1994–2013.

### Anthropometric and demographic data

For children under the age of 16 at the time of data extraction, month and year of birth are available, while, for children age 16 and over, only year of birth is recorded. The day of birth was estimated using the date of the first health record entry in the CPRD if this was in the same year and month as the recorded year and month of birth (64.5% of cases). The midpoint (15th) of the month was used as an approximation for the remaining cases with known month of birth (19.4%), while 1 July was used for the remaining cases with known year of birth only (16.1%).

The CPRD includes values of weight, height and body mass index (BMI). There may be several reasons why anthropometric data are recorded in primary care. Selection processes might introduce bias because those measured may not be representative of all registered children. When the BMI was not recorded, values were calculated from height and weight. Values for BMI were then converted into SD scores (SDS) adjusting for exact age and sex using the British 1990 growth reference data.^19^ Extreme values with SDS <−5 or >5 were removed. Only the first BMI observation per study year per child was included in the analysis, but children could contribute multiple BMI records across several study years. Overweight and obesity were defined as a BMI-SDS larger or equal to +1.04 and +1.64, respectively (equivalent to a BMI at or above the 85th and 95th centile, respectively) of the 1990 reference population.^19^

### Statistical analysis

Results are presented in three age groups (2–5 years, 6–10 years and 11–15 years) and by sex. Multilevel mixed-effects logistic regression analysis was used to examine linear trends in prevalence of overweight (including obesity) from 1994 to 2003 and 2004 to 2013. The interaction term between study year and decade was tested to investigate whether trends have changed over the two decades. Analyses take into account clustering of participants within general practices and individual children as random effects and are adjusted for age and sex as fixed effects. When fully adjusted models failed to converge, partly adjusted (ie, only for age or sex) or unadjusted models were fitted.

In order to evaluate trends over time, multilevel logistic regression analyses were conducted with overweight (vs no overweight) as the dependent variable and year of measurement as the independent variable. Analyses were repeated with obesity alone as the dependent variable to examine if trends were similar for obesity and overweight (including obesity). In all analyses, study year was the main independent variable, and the derived OR indicates the annual increase in the odds of overweight and obesity. For ease of interpretation, the annual increase is presented as percentage change in the odds of overweight and obesity (eg, 4.2% from OR=1.042). Stata v13 software was used.

## Results

In 1994 there were a total of 134 061 children aged between 2 and 15 years in the CPRD, and this increased to 479 958 in 2009 and was 427 188 in 2013. The final analysis included data from 370 544 children contributing a total of 507 483 BMI observations across the study years (average 1.4 BMI observations per child). The majority (N=287 424) contributed one observation, while the remaining contributed two to a maximum of 14 observations. Characteristics of the study sample are shown in table 1. There were 39% of the observations between 1994 and 2003 (referred to as decade 1), while the remaining 61% were taken between 2004 and 2013 (referred to as decade 2).

**Table 1:** Distribution of BMI records from 370 544 individual children in 1994–2013

	Frequency (%)
Number of BMI observations	507 483
Decade	
1 (1994–2003)	197 757 (39.0)
2 (2004–2013)	309 726 (61.0)
Sex	
Boys	259 612 (51.2)
Girls	247 871 (48.8)
Age group (years)	
2–5	136 857 (27.0)
6–10	162 976 (32.1)
11–15	207 650 (40.9)

Values are frequencies (column per cent). Data source: CPRD (Clinical Practice Research Datalink, UK).

BMI, body mass index.

### Prevalence of overweight (including obesity) between 1994 and 2013

The prevalence of overweight (including obese) is presented in table 2. Among 2–5-year-old boys, prevalence rates ranged between 19.5% (1995) and 26.0% (2007), while among 6–10-year-old boys this was between 22.6% (1994) and 33.0% (2011), and was highest among 11–15 year olds, ranging from 26.7% (1996) to 37.8% (2013). Similar findings were found for girls, with prevalence rates ranging from 18.3% (1995) to 24.4% (2008) among 2–5 year olds, from 22.5% (1996) to 32.2% (2005) among 6–10 year olds, and from 28.3% (1995) to 36.7% (2004 and 2012) in 11–15-year-old girls.

**Table 2:** Prevalence of overweight and obesity by study year, age group and sex among 370 544 children with 507 483 BMI records

	1994	1995	1996	1997	1998	1999	2000	2001	2002	2003	2004	2005	2006	2007	2008	2009	2010	2011	2012	2013
BMI records	12 676	14 377	16 100	16 719	18 684	20 197	21 398	23 407	26 058	28 141	30 142	29 952	33 898	34 848	34 420	33 316	31 673	30 241	28 914	22 322
Boys (years)																				
2–5	21.4	19.5	23.6	22.6	23.5	22.6	22.4	21.9	24.6	25.2	25.1	24.9	24.4	26.0	25.3	25.6	24.9	25.8	25.3	24.9
6–10	22.6	23.7	23.8	24.1	25.4	25.7	26.9	29.0	29.0	29.4	30.7	31.0	31.2	31.5	32.4	31.9	30.8	33.0	32.2	30.7
11–15	28.1	27.4	26.7	27.6	28.7	30.8	31.0	34.4	36.4	35.7	36.9	36.8	36.3	35.6	37.6	36.2	37.5	36.7	37.4	37.8
Girls (years)																				
2–5	19.4	18.3	20.8	19.5	21.8	22.2	21.6	21.9	21.2	22.3	23.7	23.7	22.3	23.3	24.4	22.9	22.5	24.3	23.5	23.8
6–10	24.1	24.1	22.5	24.2	23.9	25.1	25.8	27.6	29.4	29.4	31.5	32.2	30.4	31.5	31.0	30.6	30.8	30.7	31.0	30.4
11–15	29.3	28.3	28.8	29.3	31.2	32.7	32.9	33.9	34.5	35.4	36.7	35.6	34.9	36.4	35.6	35.7	36.0	35.5	36.7	36.6

Values are prevalence (%). Overweight (including obese)=BMI-SDS ≥85th centile.

BMI, body mass index; SDS, SD scores.

### Annual increases by decade

**Figure 1:**
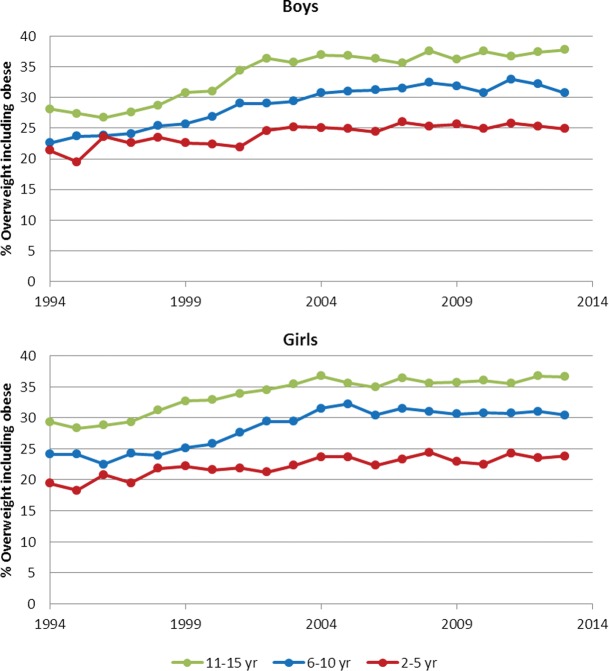
Prevalence of overweight and obesity by study year and age group in boys and girls.

Trends overtime are presented in figure 1, showing clear annual increases in the first decade (1994–2003), but less so in the second decade (2004–2013). Logistic regression models examined if changes overtime differed by decade, sex and age group (table 3). Values represent the percentage increase in odds of overweight (including obesity) per calendar year, in the full sample and in subgroups by decade, sex and age group.

**Table 3:** Percentage increase in odds of overweight and obesity per calendar year from 1994 to 2013, by decade, sex and age group

Subgroup	Number of observations	Percentage increase per calendar year*		
		Per cent	95% CI	z, p value
All	507 483	4.2	3.9 to 4.5	28.4, <0.001
Decade				
Decade 1 (1994–2003)	197 757	8.1	7.2 to 8.9	20.1, <0.001
Decade 2 (2004–2013)	309 726	0.4	−0.2 to 1.1	1.3, 0.2
Year×Decade interaction				−13.6, <0.001
Sex				
Boys	259 612	4.5	4.0 to 4.9	21.5, <0.001
Girls	247 871	3.7	3.3 to 4.2	16.8, <0.001
Year×Sex interaction	507 483			−1.2, 0.2
Boys				
Decade 1†	102 335	8.6	7.4 to 9.7	15.3, <0.001
Decade 2†	157 277	0.5	−0.5 to 1.4	1.0, 0.3
Year×Decade interaction	259 612			−10.5, <0.001
Girls				
Decade 1†	95 422	7.5	6.3 to 8.8	12.5, <0.001
Decade 2†	152 449	0.5	−0.5 to 1.6	1.1, 0.3
Year×Decade interaction	247 871			−8.4, <0.001
Age group (years)				
2–5	136 857	2.4	1.9 to 2.9	9.6, <0.001
6–10	162 976	5.1	4.5 to 5.7	17.4, <0.001
11–15	207 650	5.7	5.2 to 6.3	20.5, <0.001
Year×Age group interaction	507 483			1.7, 0.08
2–5 years				
Decade 1†	66 172	3.5	2.2 to 4.7	5.6, <0.001
Decade 2†	70 685	0.1	−1.2 to 1.5	0.2, 0.8
Year×Decade interaction	136 857			−4.5, <0.001
6–10 years				
Decade 1†	65 923	9.9	8.2 to 11.6	12.2, <0.001
Decade 2†	97 053	−1.2	−2.5 to 0.1	−1.8, 0.08
Year×Decade interaction	162 976			−10.6, <0.001
11–15 years				
Decade 1†	65 662	12.0	10.3 to 13.7	14.6, <0.001
Decade 2†	141 988	2.6	1.4 to 3.8	4.3, <0.001
Year×Decade interaction	207 650			−10.2, <0.001‡

*Analyses are based on multilevel models including level for child and practice, adjusting for age and sex. Values represent the percentage increase in odds of overweight (including obesity) per calendar year, in the full sample and in subgroups by decade, sex and age group.

†Decade 1, 1994–2003; decade 2, 2004–2013. ‡Presented results are from a model adjusted for sex only (model including age as a covariate failed to converge).

For the full sample, the odds of overweight (including obesity) increased 4.2% per study year (95% CI 3.9% to 4.5%). This annual increase differed by decade and was significantly greater from 1994 to 2003 (8.1%, 95% CI 7.2% to 8.9%) compared with 2004–2013 (0.4%, 95% CI −0.2% to 1.1%), as indicated by the significant interaction term between decade and year (p<0.001, see table 3). Annual increases over the later decade were not significant, indicating that rates of overweight (including obesity) are stabilising in the full sample of children across age groups and both sexes.

### Annual increases by sex

Annual increases did not differ by sex as indicated by the non-significant interaction between study year and sex (p=0.244). Both in boys and girls, the annual increase was significant in the first decade (8.6% and 7.5%, respectively) but not in the later decade (0.5% in both sexes, similar to findings in the full sample; see table 3).

### Annual increases by age group

Annual increases differed by age group as indicated by the borderline significant interaction between study year and age group (p=0.08), and showed smaller increases in the younger age group (age 2–5 year: 2.4%, 95% CI 1.9% to 2.9%) compared with the older age groups (5.1% and 5.7%, respectively, in 6–10 year olds and 11–15 year olds). When the annual increases were analysed by age group, results showed that increases were significant in all age groups in decade 1 (1994–2003), but annual increases were not significant in decade 2 (2004–2013) in the younger (2–5 year) and middle (6–10 year) age groups. Increases were still statistically significant for the older age group (11–15 year) in decade 2, albeit significantly smaller compared with the first decade (1994–2003: 12.0%, 95% CI 10.3% to 13.7%; 2004–2013: 2.6%, 95% CI 1.4% to 3.8%), as indicated by the significant interaction term (p<0.001).

### Obesity

**Figure 2:**
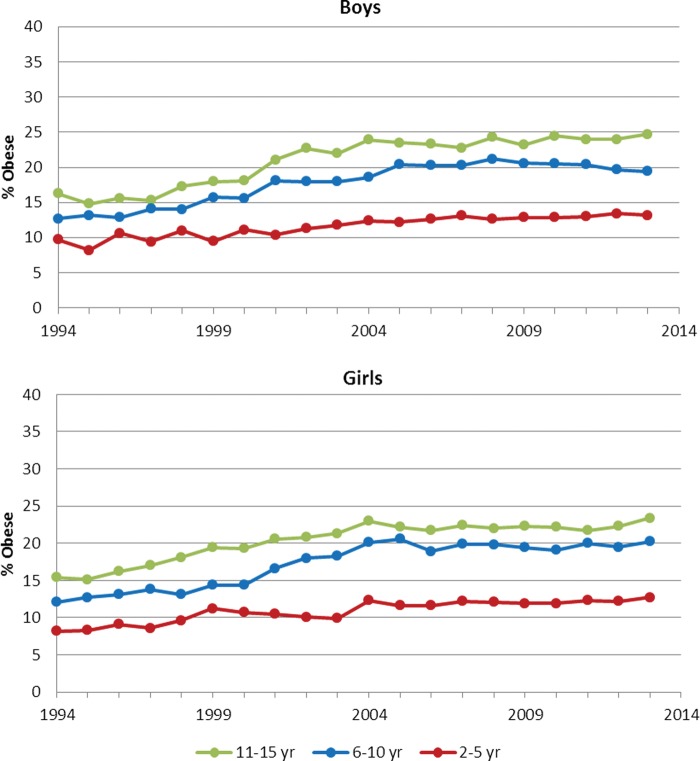
Prevalence of obesity by study year and age group in boys and girls.

The prevalence of obesity increased over the study period (figure 2), in all three age groups in both sexes. Estimated trends were comparable to those shown for overweight (including obese), indicating significant increases in the first decade but no significant trends in the later decade (data not shown). Again trends were similar for boys and girls, but differed by age groups, with obesity prevalence stabilising in 2004 to 2013 in the younger (2–10 year) but not older (11–15 year) age group, where obesity rates continued to increase.

## Discussion

This study contributes to proof-of-concept for use of primary care electronic health records to evaluate children's obesity in England and suggests these may provide a valuable resource for monitoring obesity trends. Observed trends in childhood obesity are dramatically different between decades, with an annual 8% increase during 1994–2003 and a small 0.4% annual increase during 2004–2013, which is not a significant increase despite the large numbers included in the analyses. Our findings also indicate that only among 11–15 year olds does prevalence still increase, whereas in younger ages annual increases stabilised between 2004 and 2013. Observed prevalence rates of overweight and obesity and trends in the CPRD are consistent with those reported in the HSE^15^ and NCMP as noted above.^16^ No obvious differences between sexes were identified,^8^ although data from the HSE suggest that a downward trend occurred in girls.^20^

There are several possible theories for the recent stabilisation of childhood overweight and obesity rates. One explanation may be that rates have reached a point of saturation.^7^ The cumulative effect of public health campaigns may now be resulting in stabilisation of prevalence of overweight and obesity in children. Lastly, an alternative explanation may be that it is an artefact from the electronic data used in our study. However, similar trends have been reported for other data sources and countries.^7^ ^8^

### Strengths and limitations

The study has shown that the CPRD includes a large number of BMI records across the full age range of children. This is important in terms of generalisability and validity to draw conclusions on a national level. It also allowed us to study trends in subgroups according to age and sex with confidence, as numbers were large even in the subgroups. The CPRD practices, and the CPRD registered population, are considered to be sociodemographically representative of the UK population.^17^ ^21^ We only included the CPRD practices in England that participate in the data linkage scheme. The registered population for these practices generally shows a similar regional distribution to the resident population for England, although there is a slightly lower proportion in Yorkshire and Humber and a slightly greater proportion in the South East region. CPRD data generally have high validity,^18^ but the validity of height and weight values measured by practice staff requires evaluation.

Participation in terms of percentage of children with measurements in each study year may be low, as very few children have their BMI recorded every year. A limitation is that BMI recording might be biased by confounding by indication, and that BMI is recorded when children are ill, or in those with particularly low or high body weights. Test recording in the CPRD has increased over time, as have concerns with children's obesity, and the latter may influence the propensity for BMI values to be recorded. However, analyses have shown that children who contributed observations to multiple study years did not differ on BMI-SDS compared with those with BMI recorded in only a single study year, and our results were comparable to those observed in other UK datasets (HSE and NCMP).^15^ ^16^ We acknowledge that additional non-linear modelling might contribute to a better understanding of the deceleration in trend, and whether processes might differ across age groups. Ethnic differences in overweight and obesity prevalence are well established, but the CPRD does not include comprehensively recorded data on ethnicity. Socioeconomic differentials in obesity are documented, but there is potential for future research to examine whether socioeconomic differences are consistent in all ages and both sexes.

## Conclusion

Primary care electronic health records in England may provide a valuable resource for monitoring obesity trends and may be used to supplement existing surveillance systems. Our data suggest that routine growth monitoring of children may not always be captured into electronic records; improved data recording will increase the utility of this data resource. This study shows that overweight and obesity prevalence in the UK increased during decade 1 (1994–2003) but stabilised in decade 2 (2004–2013). This was observed in both sexes and in the younger age groups (2–5 years and 6–10 years). However, rates continued to increase in older children (11–15 years), albeit at a slower speed than in decade 1 (1994–2003). Increasing the recognition of obesity in primary care, and the effectiveness of interventions delivered through primary care services, represents one important component of the overall policy response.
